# Adolescence in crisis: Insights from the 2024 ESPAD report and a nursing perspective

**DOI:** 10.1016/j.isci.2026.114726

**Published:** 2026-01-19

**Authors:** María José Ferreira Díaz

**Affiliations:** 1 Faculty of Nursing, University of Santiago de Compostela, Campus Lugo, Lugo, Spain

**Keywords:** health sciences, medicine, medical science, public health, patient social context

## Abstract

Around the world, adolescents are facing unprecedented levels of psychological distress, loneliness, and digital saturation. Data from the *ESPAD 2024* survey, WHO’s *Global Health Estimates*, and UNICEF’s *State of the World’s Children* reveal a consistent decline in subjective well-being and a surge in anxiety, self-harm, and substance use. Despite an explosion of mental-health advocacy, the everyday environments where adolescents live and learn remain poorly equipped to foster resilience and belonging. This paradox—rising awareness but weakening support—marks a critical failure of preventive healthcare systems. By linking the 2024 ESPAD evidence with nursing science and educational reform, this perspective offers a methodologically rigorous and globally transferable model to address the adolescent well-being crisis through preventive, person-centered care.This *perspective* argues that the adolescent well-being crisis is not primarily a psychiatric phenomenon but a social and educational one. The dominance of therapeutic and pharmacological responses has obscured the preventive and relational dimensions of care. Nursing, with its foundations in empathy, communication, and community engagement, offers a pathway toward a new preventive paradigm—one that unites evidence and humanity. Drawing on nursing taxonomies (NANDA-I, NIC, and NOC) and contemporary pedagogical frameworks, this article reframes prevention as an educational and ethical responsibility that extends beyond clinical boundaries.By integrating insights from epidemiology, pedagogy, and nursing science, the article calls for a human-centered transformation of adolescent health policy and professional education. Empowering nurses to lead preventive action can bridge the gap between public-health data and lived experience, restoring emotional literacy, connection, and purpose among young people. The next revolution in adolescent mental health will not be purely technological or therapeutic—it will be profoundly human.

## Graphical abstract

**Figure:**
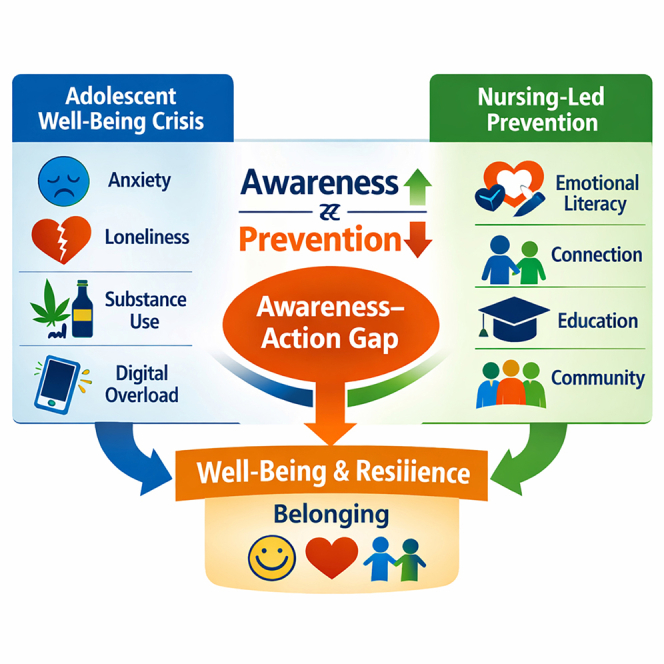

## Introduction

**Figure 1:**
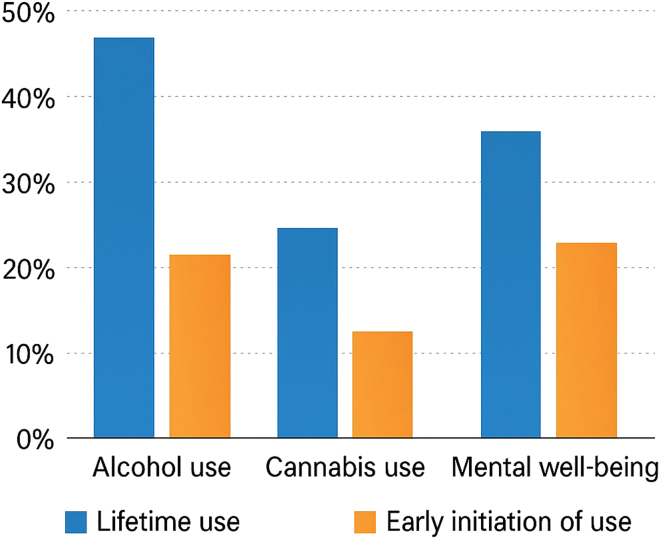
Adolescent substance use and perceived well-being according to the 2024 ESPAD report Percentage of European adolescents reporting lifetime use and early initiation of alcohol and cannabis, together with self-reported levels of mental well-being. Data adapted from the ESPAD report 2024 (European Monitoring Center for Drugs and Drug Addiction).

Across the world, adolescence has become one of the most vulnerable phases of human development. Data from the *European School Survey Project on Alcohol and Other Drugs (ESPAD 2024)* show that more than one in four European adolescents report low life satisfaction, while rates of anxiety, self-harm, and substance use continue to rise (Figure 1).^1^ Globally, the *World Health Organization* (WHO) estimates that depression and anxiety are now among the top five causes of disability in adolescents, and suicide remains the second leading cause of death among people aged 15–19 years.^2^ According to *UNICEF’s State of the World’s Children 2024*, young people are facing an “epidemic of disconnection,” characterized by loneliness, academic pressure, and digital overexposure.^3^

Paradoxically, this crisis unfolds at a time of unprecedented awareness of mental health. Governments, media, and social platforms now promote psychological well-being as a universal priority, yet the daily contexts in which adolescents live—families, schools, and communities—remain poorly equipped to sustain emotional resilience. *Science* recently described this gap as the “mental-health paradox”: a global expansion of advocacy without a parallel investment in prevention or care.^4^

This *perspective* argues that the adolescent mental-health crisis is not primarily psychiatric but profoundly social and educational. While clinical interventions remain essential, they often arrive too late and focus narrowly on symptom management. What is missing is a preventive, relational infrastructure capable of identifying distress early and nurturing emotional literacy. Nursing, with its foundations in empathy, communication, and continuity of care, is uniquely positioned to bridge this divide. Within community and school settings, nurses can translate epidemiological data into actionable prevention, supporting self-care, coping skills, and social connection.^5^

Addressing this challenge demands more than expanding access to mental-health services; it requires reimagining how societies educate, support, and relate to their youth. As the *Lancet Commission on Health Professionals for a New Century* emphasized, professional education must be transformative, preparing health workers not only to treat disease but to lead social change.^6^ Reframing adolescent well-being as a shared public-health responsibility will redefine the boundaries of prevention and restore the human dimension at the heart of care.

While existing public-health and school-based mental-health models emphasize risk surveillance, screening, or referral pathways, they rarely address the relational and educational foundations needed to sustain adolescents’ emotional well-being. Current frameworks tend to focus on clinical indicators or symptom-based algorithms, leaving limited space for preventive competencies such as emotional literacy, connection, and everyday caregiving. The preventive paradigm proposed in this Perspective diverges from these models by positioning nursing—particularly community and school-based nursing—as the professional field best equipped to integrate epidemiological evidence with relational, educational, and context-sensitive care. This human-centered approach complements existing structures while addressing their most persistent gaps: the lack of preventive infrastructures and the insufficient integration of emotional competencies within health and educational systems.

### Methodological framing of evidence integration

To support the conceptual argument developed in this perspective, three epidemiological datasets were selected based on their methodological rigor, cross-national comparability, and relevance to adolescent well-being: the ESPAD 2024 survey, the WHO Global Health Estimates, and UNICEF’s State of the World’s Children report. These sources were chosen because they provide harmonized indicators across multiple countries, longitudinal continuity, and complementary perspectives on behavioral risks, mental distress, and social determinants. Their triangulation enables a coherent interpretation of trends showing increasing psychological vulnerability despite rising mental-health awareness. This synthesis underpins the “awareness–action paradox,” highlighting how epidemiological visibility has not translated into preventive infrastructures capable of supporting adolescents in their everyday environments.

## Integrative significance statement

This perspective addresses a global public-health challenge grounded in robust evidence: the deterioration of adolescent well-being documented in the 2024 ESPAD^1^ report and corroborated by WHO^2^ and UNICEF^3^ data. It identifies a clear gap between the growing visibility of mental-health advocacy and the persistent weakness of preventive infrastructures. By integrating epidemiological evidence with nursing science and educational innovation, the article proposes a human-centered framework that redefines prevention as a relational and ethical act.

Beyond describing the problem, it offers a transferable model for action—linking global data to pedagogical strategies that prepare nurses to anticipate risk, foster emotional literacy, and strengthen resilience in community and school settings. The article thus combines scientific rigor, conceptual novelty, and practical relevance: it advances nursing as a transformative discipline capable of operationalizing prevention, bridging evidence and empathy, and aligning health education with person-centered, context-sensitive care.

## The paradox of awareness: When mental health advocacy outpaces prevention

Over the past decade, mental health has become one of the most visible causes in global health policy and media discourse. Governments invest in awareness campaigns, celebrities become advocates, and hashtags mobilize millions around the importance of “talking about mental health.” Yet despite this visibility, the lived reality of adolescents tells a different story. Suicide rates remain stubbornly high, and reported symptoms of depression, anxiety, and loneliness continue to escalate across continents.^1^ The paradox is striking: the louder the conversation grows, the weaker the structures of prevention appear to be.

This “awareness-action gap” reflects a cultural and institutional imbalance. Awareness is politically appealing—it signals empathy without demanding structural change. Prevention, on the other hand, is slower, relational, and rarely visible. It requires time, training, and sustained human presence, elements that modern systems of education and healthcare increasingly undervalue. As a recent *Science* commentary noted, societies have become “diagnostically fluent but relationally impoverished,” recognizing distress but lacking the mechanisms to respond.^2^

The overemphasis on awareness also fuels a subtle medicalization of adolescence. Behaviors once understood as part of developmental turbulence—sadness, restlessness, and self-doubt—are increasingly labeled as pathological. While early recognition of genuine mental disorders is crucial, an overreliance on diagnostic frameworks risks replacing education and community support with pharmacological solutions.^3^ This diagnostic inflation narrows our collective imagination about what care can mean.

The nursing perspective offers an alternative logic. Instead of reducing adolescents to patients in waiting, it views them as developing persons embedded in social contexts. Nurses, especially those in community and school health, are trained to detect early signs of distress not through symptom checklists but through presence, listening, and engagement.^4^ Their preventive interventions—guided by frameworks such as NANDA-I, (*NANDA International Nursing Diagnoses*), NIC (*Nursing Interventions Classification*), and NOC (*Nursing Outcomes Classification*)^7^—translate empathy into structured action, linking emotional literacy, peer support, and behavioral self-regulation. This combination of evidence and humanity embodies what public health has been missing: a relational infrastructure of prevention.

Ultimately, the paradox of awareness invites a new question: what if the next great innovation in adolescent mental health is not technological but human? The answer may depend less on inventing new treatments than on rediscovering the everyday spaces—schools, clinics, and communities—where care and connection can flourish again.

## Beyond therapy: Building preventive infrastructures

If the first paradox of our time is that awareness has expanded faster than prevention, the second is that health systems remain structurally reactive. They intervene once distress becomes disorder, once vulnerability becomes crisis. This reactive logic—rooted in biomedical thinking—has shaped not only how we treat mental illness but how we educate health professionals. Training programs still prioritize clinical management over prevention, leaving little room for emotional literacy, social determinants, or community-based competencies,^1^ reinforcing the need to integrate research and preventive competences throughout nursing education.^6^

### Addressing current gaps in policy and education

Despite increasing recognition of adolescent mental distress, existing mental-health policies and educational frameworks remain largely reactive and clinically oriented. Most public-health strategies emphasize risk screening, diagnostic categorization, or referral pathways, while school and professional curricula continue to prioritize cognitive mastery over relational and preventive competencies. As noted in international analyses of health-professional education, these curricula often fail to incorporate the transformative and preventive capacities required by contemporary public-health challenges.^4^^,^^5^ As a result, emotional literacy, early identification of distress, and the everyday relational labor that sustains well-being remain insufficiently embedded in training and practice.^8^ The paradigm proposed in this perspective directly addresses these gaps by operationalizing preventive nursing taxonomies (NANDA-I, NIC, and NOC) within educational and community settings. By framing prevention as a relational, evidence-informed, and context-sensitive practice, this approach offers a complementary model to current policies—one capable of transforming the environments where adolescents actually live, learn, and struggle.

Building a preventive infrastructure requires reorienting both education and practice. Prevention is not an abstract ideal; it is an operational capacity that must be designed, taught, and sustained. Nurses are among the few professionals trained to think and act across this continuum—from individual to community, from symptom to system. Their interventions, as defined by NIC and NOC frameworks, explicitly address coping, resilience, and social connection.^7^ These are not peripheral skills; they are the emotional technologies of public health.

However, prevention cannot thrive in isolation. It needs an ecosystem that links evidence, pedagogy, and institutional will. Integrating adolescent well-being into nursing and public-health curricula is an urgent step. Educational innovation—through simulation, service learning, and reflective practice—can translate population data into lived competencies,^8^ fostering emotional competence and reflective capacity in nursing students.^7^ In Spain, for example, university programs are beginning to incorporate experiential modules on school-based health promotion, allowing nursing students to design interventions grounded in real community contexts. Similar approaches are emerging across Europe and Latin America, where prevention is taught as both a technical and ethical responsibility.^4^

The challenge is not simply to add new content to existing curricula but to reimagine the purpose of professional education. As Frenk et al. argued in the *Lancet Commission on Health Professionals for a New Century*, the ultimate goal of health education is social transformation.^6^ This transformation begins when prevention is understood as a collective act of care—one that bridges epidemiology and empathy, data and daily life.

A preventive infrastructure for adolescent well-being must therefore operate on three levels.

(1) Knowledge, grounded in public health and behavioral evidence;
(2) Competence, developed through active, relational pedagogy; and
(3) Culture, nurtured by institutions that value human connection as a measurable outcome.

Only by aligning these levels can societies move beyond therapy toward a sustainable, preventive model of mental health.

## Educating for human connection

### Recognizing deficits in current nursing education

Despite the growing emphasis on mental health and prevention, many nursing programs still integrate relational, emotional, and community-based competencies only superficially or inconsistently. International analyses have shown that curricula continue to privilege biomedical content, technical skills, and acute-care competencies, leaving limited space for emotional literacy, reflective practice, or preventive approaches to adolescent well-being.^4^^,^^5^ Furthermore, recent educational research highlights that emotional competence remains underdeveloped among nursing students, partly due to the lack of structured pedagogical strategies to cultivate it.^8^ Acknowledging these gaps underscores the urgency of reorienting nursing education toward preventive, relational care—an orientation that aligns with the human-centered paradigm proposed in this perspective.^9^

If prevention is to become a new foundation for adolescent health, it must be taught not only as a public-health strategy but as a human discipline. Education is where empathy becomes a skill and compassion becomes a method. Yet most health curricula still privilege cognitive mastery over emotional competence, treating communication, self-reflection, and relational awareness as “soft” skills rather than core components of professional identity.^6^

Rebalancing this hierarchy is essential. The nursing profession has long understood that knowledge detached from human connection loses its transformative power. Training students to recognize suffering, listen without judgment, and respond authentically is not sentimental pedagogy—it is evidence-based prevention. Emotional literacy reduces burnout, improves clinical communication, and enhances patient outcomes.^8^ For adolescents, it represents the difference between being treated and being understood.

Educational research in nursing shows that experiential learning—simulation, peer teaching, and service-learning projects—can cultivate precisely these capacities.^3^^,^^8^ When students engage with real communities, they begin to see prevention not as a theoretical construct but as a lived relationship. Reflection, guided by educators, turns experience into insight. In this process, the classroom becomes a microcosm of society: a space where care is practiced, not merely discussed.

Technology can assist but not replace this process. Virtual platforms may extend access, but human connection remains the medium through which empathy and ethical reasoning are transmitted. As Patel recently argued in *Science*,^4^ “*the future of mental health will depend less on the reach of our algorithms than on the reach of our attention*.” Teaching future nurses to attend—to be present, embodied, and relational—is thus an act of resistance against the mechanization of care.

Restoring human connection within health education is not nostalgic; it is revolutionary. It reclaims prevention as a cultural practice and redefines professionalism as the capacity to care intelligently and compassionately. By educating for connection, nursing can help rebuild the social fabric that adolescent well-being depends on.

### Strengthening the evidentiary basis for educational reform

The limited integration of emotional literacy and relational competencies within current health-professional education is well documented. Systematic reviews show that nursing curricula continue to prioritize technical and biomedical content, with insufficient attention to experiential learning, reflective practice, or the development of emotional competence.^8^ Empirical studies further demonstrate that nursing students frequently report low levels of emotional awareness and limited preparation for relational care, particularly in adolescent and community settings.^8^ European curricular analyses likewise underline persistent gaps in preventive and research-related competencies, indicating a broader structural misalignment between training programs and the psychosocial needs of young people.^6^ These findings substantiate the claim that emotional and relational skills remain undervalued within existing educational frameworks and reinforce the need for the preventive, human-centered orientation proposed in this Perspective.

### Limitations of this perspective

This perspective is grounded primarily in European epidemiological datasets, including ESPAD, WHO, and UNICEF reports, which may limit the generalizability of specific patterns of adolescent well-being to other cultural or socioeconomic contexts. The conceptual framework proposed here is also based on secondary data and theoretical integration rather than primary empirical research. As such, the recommendations presented may require contextual adaptation in settings with different educational structures, community resources, or nursing roles. These limitations are inherent to the Perspective format, whose purpose is to synthesize evidence, identify gaps, and outline a preventive, human-centered paradigm that can inform—but not replace—locally grounded empirical inquiry.

## Conclusion: A human-centered revolution in adolescent health

The global crisis of adolescent well-being demands more than improved diagnostics or greater access to therapy; it calls for a fundamental reorientation of how societies understand health itself. Mental distress among young people is not an isolated clinical issue but a mirror reflecting the erosion of social connection, empathy, and shared purpose. Addressing it will require rebuilding what modern systems have neglected: the human infrastructures of prevention.

Nursing embodies precisely the competencies that can guide this transition. As a discipline, it bridges science and humanity, data and compassion. The preventive mindset embedded in nursing practice—early detection, communication, and empowerment—offers a transferable framework for restoring equilibrium to health systems overwhelmed by crisis management.^7^ Nurses do not merely intervene in illness; they sustain the everyday conditions of well-being. Recognizing and amplifying this role is therefore not only a professional necessity but a societal imperative.

The next revolution in adolescent mental health will not emerge solely from new molecules, devices, or apps. It will emerge from a collective rediscovery of care as a form of intelligence: the capacity to notice, to connect, and to act before harm becomes irreversible. Crucially, this human-centered paradigm is not opposed to clinical or technological innovation; rather, it complements and strengthens them by grounding mental-health interventions in the relational and preventive foundations that adolescents rely on. Integrating this logic into education, policy, and community practice would redefine what progress in health means.

Public health, at its best, is an act of solidarity. By aligning the scientific and the humane, we can move from awareness to accompaniment, from therapy to prevention, and from isolation to belonging. In this shift, nursing can serve as both compass and catalyst—a profession reminding us that prevention is not only about avoiding disease, but about protecting the fragile networks of meaning that sustain life itself in line with evidence that youth social connectedness is a fundamental determinant of mental health.^10^

## From traditional risk factors to emerging realities in nursing education

For decades, preventive nursing education has been structured around the “classic triad” of behavioral risks: alcohol, tobacco, and illicit drugs. These categories shaped not only public-health campaigns but also the mindset of nursing students trained to identify and manage tangible, substance-related harms.^1^ However, the social landscape of adolescence has changed profoundly. Today’s young people navigate a hybrid reality in which digital exposure, academic pressure, emotional fatigue, and loneliness have become equally powerful determinants of health.^2^

This shift requires an educational response as transformative as the challenge itself. Nursing curricula must evolve from risk surveillance to resilience cultivation, from detecting pathology to promoting well-being. The ESPAD 2024 report provides a data-driven roadmap for this transition: while traditional behaviors remain relevant, the report underscores the rising influence of digital risks, psychosocial stress, and declining emotional support (Table 1).^3^

**Table 1:** From traditional risk factors to emerging priorities in adolescent health and nursing education

Health domain	Traditional focus (pre-2020)	Emerging priorities (post-ESPAD 2024)	Relevant nursing taxonomies	Educational strategy
Substance use	Alcohol, tobacco, cannabis	Polysubstance use, vaping, early initiation	NANDA-I 00178 Risk for Substance Use; NIC 4500 Substance Use Prevention	Motivational interviewing; peer-education projects
Emotional well-being	Anxiety and depression screening	Emotional literacy, stress regulation, mindfulness	NANDA-I 00069 Ineffective Coping; NOC 1402 Emotional Control	Simulation and reflective journaling
Social interaction	Bullying, peer pressure	Loneliness, digital isolation, online vulnerability	NANDA-I 00052 Impaired Social Interaction; NIC 5440 Coping Enhancement	Group facilitation; service-learning with schools
Digital health	Not systematically addressed	Screen-time balance, cyber risks, digital self-care	NANDA-I proposed Risk for Impaired Digital Well-being; NIC 5510 Health Education	Critical analysis of digital media; digital-literacy modules
Gender and inclusion	Limited gender focus	Gender-sensitive mental-health support, equity in care	NANDA-I 00168 Risk for Powerlessness; NOC 1503 Social Involvement	Inclusive communication training; advocacy exercises

Overview of how nursing education can integrate epidemiological evidence with pedagogical strategies addressing both traditional risk behaviors (alcohol, tobacco, and illicit drugs) and emerging psychosocial and digital risk domains among adolescents.

Integrating these dimensions into training is not an optional modernization—it is an ethical duty. Nurses must now be prepared to understand the dynamics of screen addiction, social-media anxiety, and virtual peer influence, while still addressing substance use and mental distress. This expanded competence framework aligns with the preventive philosophy of the NANDA-I, NIC, and NOC taxonomies,^7^ which position emotional literacy and social connectedness as measurable outcomes of care.^4^

## Acknowledgments

The author wishes to thank the European School Survey Project on Alcohol and Other Drugs (ESPAD) research teams and participating schools for their valuable contribution to adolescent health data and prevention research.

This research did not receive any specific grant from funding agencies in the public, commercial, or not-for-profit sectors.

## Declaration of interests

The author declares no known competing financial interests or personal relationships that could have appeared to influence the work reported in this paper.
